# Productive and reproductive performances of dairy cattle herds in Treviso province, Italy (2009–2012): an assessment of the potential impact of Schmallenberg virus epidemic

**DOI:** 10.1186/s12917-015-0527-1

**Published:** 2015-08-11

**Authors:** Marica Toson, Lapo Mughini-Gras, Katia Capello, Laura Gagliazzo, Laura Bortolotti, Matteo Mazzucato, Stefano Marangon, Lebana Bonfanti

**Affiliations:** Istituto Zooprofilattico Sperimentale delle Venezie (IZSVe), Viale dell’Università 10, 35020 Legnaro, (Padua) Italy; National Institute for Public Health and the Environment (RIVM), Centre for Infectious Disease Control (CIb), PO Box 1–3720, BA Bilthoven, The Netherlands

**Keywords:** SBV, Orthobunyavirus, Dairy cattle, Italy

## Abstract

**Background:**

Schmallenberg virus (SBV) has spread across Europe since mid-2011, causing unspecific and transitory symptoms in ruminants and congenital malformations in their offspring. Evidence for the impact of SBV on cattle (re)productive performance is limited. Using a comprehensive data set from a SBV-affected province in North-East Italy, this study aimed at assessing the potential impact of SBV emergence on 11 productive and reproductive performance indicators of dairy cattle herds, accounting for weather conditions and other herd-level factors that could also influence these indicators.

**Results:**

A total of 127 farms with an average of 71 cows per farm (range 29–496) were monitored monthly from January 2009 to June 2012. Mixed-effects linear models for longitudinal data were used to assess the average variation in herds’ performance indicators over semesters (Jan-Jun 2009, Jul-Dec 2009, Jan-Jun 2010, Jul-Dec 2010, Jan-Jun 2011, Jul-Dec 2011, Jan-Jun 2012) and trimesters therein. Taking the second semester of 2011 as reference, significant decreases in the average lactation length (−6 days, on average) and calving-to-conception interval (−4 days, on average) were observed relative to the same semesters of the years 2010 and 2009, respectively. Similarly, during the last trimester of 2011, which is most likely to cover the SBV infection period in the study area, there was an average decrease of −4 days (lactation length) and −7 days (calving-to-conception interval) compared to the same trimesters of the years 2010 and 2009, respectively. However, the observed decreases actually represent a positive outcome that is not as such imputable to SBV emergence, but rather reflects other beneficial changes in farm management. None of the other indicators showed significant variations, confirming the relatively mild expression of SBV infection in cattle.

**Conclusions:**

Although the emergence of SBV might have significantly affected the (re)productive performance of some individual farms, we concluded that overall at the province level there were no significant variations attributable to SBV, at least not in a way that would lead to negative effects on farm profitability.

**Electronic supplementary material:**

The online version of this article (doi:10.1186/s12917-015-0527-1) contains supplementary material, which is available to authorized users.

## Background

Schmallenberg virus (SBV), a newly discovered *Orthobunyavirus* (family *Bunyaviridae*), was first identified in November 2011 from dairy cattle with unspecific and transitory symptoms, including fever, decreased milk production and diarrhoea, in North Rhine-Westphalia, Germany [1]. Schmallenberg virus shows high homology to viruses of the Simbu serogroup, the members of which are typically transmitted through *Culicoides* biting midges [2, 3] and can cause congenital defects in domestic animals [4, 5].

The first clinical evidences of SBV appeared in August 2011, not only in Germany, but also in Belgium and the Netherlands. Because examination of archived samples did not indicate prior SBV circulation, the most accredited hypothesis is that the virus was introduced in Europe between spring and summer 2011, and that the excess of congenital malformations, abortions, and stillborns that have been reported in domestic ruminants since December 2011 are the result of SBV infections in pregnant animals between summer and autumn 2011 [6]. By May 2013, a total of 8730 bovine and ovicaprine herds in 22 European countries, including Italy, were reported as being SBV-positive based on laboratory confirmation of suspected cases [7].

**Fig. 1:**
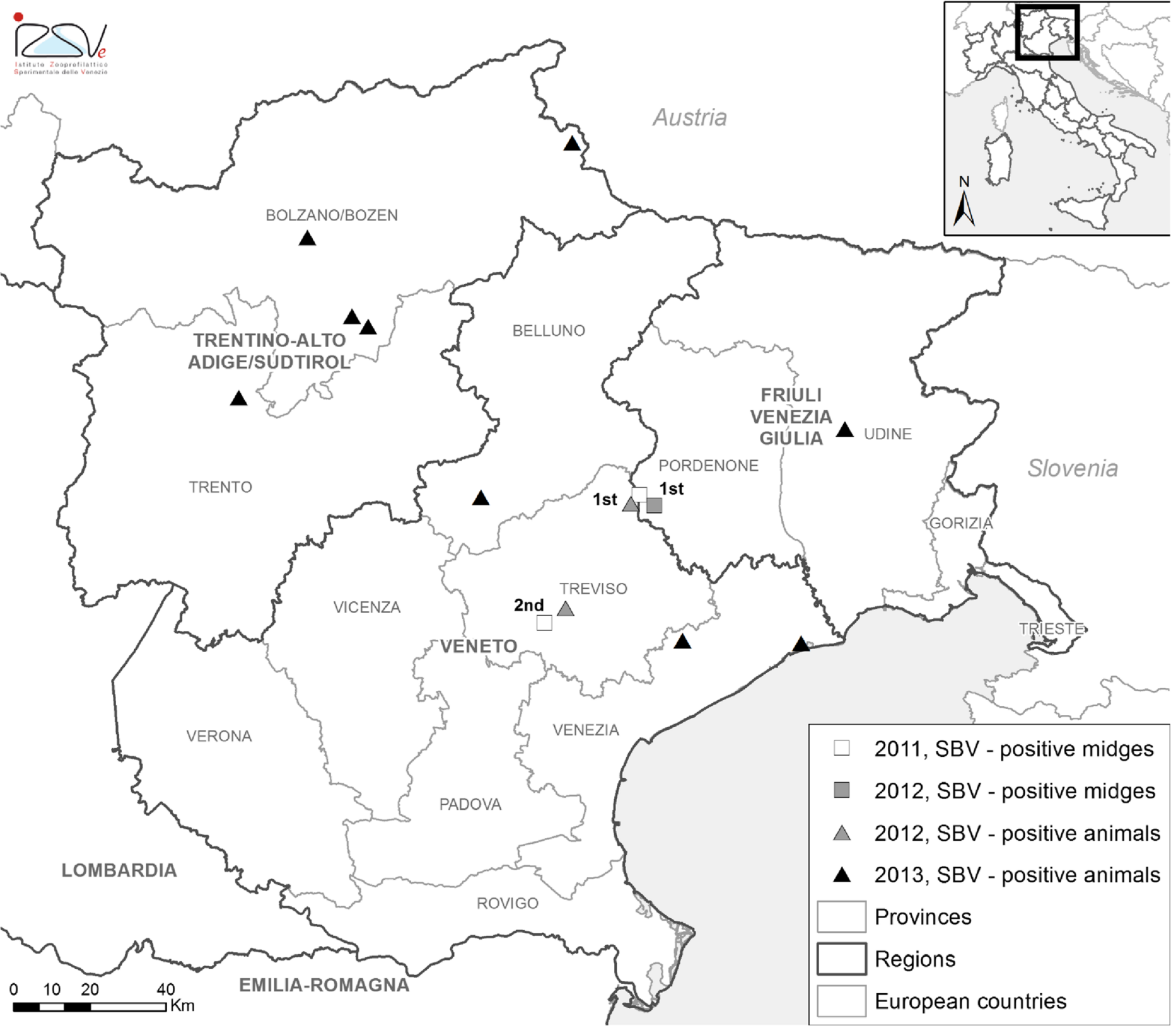
Map of North-East Italy, including the study area (Treviso province), with indication of positivities for Schmallenberg virus in *Culicoides* biting midges and susceptible animals (affected farms) from 2011 to 2013. The first SBV-positive animal was detected in Treviso province in a dystocic goat on the first semester of 2012 (1st). Midges were found positive in Treviso province on the second semester of 2011 (2nd) and in Pordenone province on the first semester of 2012 (1st)

In Italy, the first laboratory-confirmed SBV case was reported at the beginning of February 2012 from a small multi-species farm in the province of Treviso, Veneto region (Fig. 1), where a dystocic goat died because of the retention of a malformed foetus [8]. Epidemiological investigation around this farm excluded any prior introduction of animals from other European countries, suggesting that the virus had circulated locally. Moreover, six pools of *Culicoides* midges collected as part of the national surveillance programme for bluetongue virus (BTV) between September and November 2011 within a 50 km radius from the first SBV case tested positive for SBV [2, 8]. This provided evidence that SBV had circulated in the local vector population almost five months before detecting the first case, but that any circulation before September 2011 was unlikely [2, 8].

Evidence for the impact of SBV on animal (re)productive performance is scarce. A few studies have assessed the impact of SBV infection as: 1) direct impact on adult animals, including their clinical manifestations and herd- or area-level variation in key performance parameters [9–11]; 2) direct impact on newborns, including congenital malformations, abortions, and stillbirths [7, 9, 12]; and 3) indirect impacts, including trade restrictions and treatment costs [9, 13]. Although SBV may cause clinical signs of pyrexia and reduced milk production, cases are typically identified only when animals appear with congenital malformations among offspring whose mothers acquired infection during a particular period of pregnancy [9]. The lack of a clear clinically overt presentation is one of the main reasons as to why there is a large degree of under-ascertainment for SBV [7], especially in cattle, for which it is known that SBV infection is relatively mild, with a low incidence of birth malformations [7, 9–11]. Accordingly, the impact of SBV on adverse pregnancy outcomes (abortions, stillbirths, and congenital malformations) of dairy cattle is believed to be limited and lower than that on milk production or return to service [6]. A recent Dutch study reported that calf mortality differed very little between herds with low and high SBV seroprevalence during the second half of 2011, a finding that mirrors the relatively mild expression of SBV infection in adult cows [10]. Conversely, SBV seemed to have had a relatively more pronounced impact on milk production. In a predefined 4-week period during which most SBV infections were expected to have occurred in the Netherlands, the overall drop in milk production per cow was quantified as ~30 kg (in herds with high SBV seroprevalence) and ~50 kg (in herds with reported SBV-associated symptoms) [10]. However, potential confounders such as weather conditions, particularly temperature and relative humidity, which are known to affect milk production levels [14], were not accounted for.

This study aimed at assessing variations imputable to SBV emergence in 11 productive and reproductive performance indicators of dairy cattle herds in the province of Treviso, i.e. Italy’s area where SBV was at first detected [8] and post-epidemic modelling studies have predicted it as one of the areas with the highest risk of SBV spread in Europe [15]. The analysis accounted for weather conditions and other herd-level factors that could also influence these indicators. Yet, it could only assess the presumptive/circumstantial effect of SBV on dairy cattle herd performances since no exact SBV data were available for the study area.

## Results

The productive and reproductive performance indicators of dairy cattle herds in the study area are summarized by semester in Table 1. Data were collected monthly from January 2009 to June 2012 at the herd level (*n* = 127 herds). The average number of herd-month observations by semester per each indicator varied from 591 (Jan-Jun 2012) to 726 (Jan-Jun 2012), depending on the number of missing values. Some indicators (i.e. average daily milk production and total number of registered newborns) showed a clear seasonal pattern, with a tendency towards decreased milk production in the second semester (Jul-Dec) of each year (which comprises the summer) and increased number of newborns in the first semester (Jan-Jun) of each year (which comprises the spring). Accordingly, the average number of inseminations per pregnancy leading to a healthy newborn increased in the second semester of each year. The indicator average lactation length showed a drop in Jul-Dec 2011, which was preceded in first half of 2011 by another drop as compared to the previous semesters. The same was observed for the average calving-to-conception interval.

**Table 1:** Mean values (±standard deviation) of the productive and reproductive performance indicators of dairy cattle herds of Treviso province, North-East Italy. Data were collected monthly at the herd level (*n* = 127 herds), from January 2009 to June 2012

Herd-level performance indicators		Semesters						
		Jan-Jun 2009	Jul-Dec 2009	Jan-Jun 2010	Jul-Dec 2010	Jan-Jun 2011	Jul-Dec 2011	Jan-Jun 2012
		(*n* = 710)	(*n* = 620)	(*n* = 726)	(*n* = 636)	(*n* = 747)	(*n* = 632)	(*n* = 591)
1	Average daily milk production per cow (L/day/cow)	29.08 (±5.46)	27.44 (±4.54)	29.23 (±4.52)	27.05 (±4.57)	29.16 (±4.69)	27.33 (±4.60)	29.32 (±4.69)
2	Average milk protein content (%)	3.43 (±0.19)	3.43 (±0.16)	3.39 (±0.14)	3.4 (±0.28)	3.36 (±0.14)	3.43 (±0.18)	3.42 (±0.15)
3	Average milk fat content (%)	3.65 (±0.33)	3.73 (±0.32)	3.67 (±0.31)	3.71 (±0.43)	3.61 (±0.32)	3.68 (±0.32)	3.72 (±0.30)
4	Average number of lactations per cow (no.)	2.37 (±0.30)	2.36 (±0.30)	2.34 (±0.28)	2.34 (±0.27)	2.33 (±0.27)	2.33 (±0.28)	2.33 (±0.28)
5	Average lactation length (days)	200.75 (±34.37)	203.41 (±37.20)	203.42 (±38.30)	203.83 (±38.73)	198.8 (±33.28)	195.03 (±32.89)	198.89 (±31.81)
6	Average calving-to-conception interval (days)	163.65 (±34.65)	167.28 (±38.08)	164.03 (±38.36)	166.25 (±38.95)	159.34 (±34.71)	158.44 (±34.60)	157.5 (±34.46)
7	Average number of inseminations per pregnancy (no.)	2.38 (±0.63)	2.36 (±0.66)	2.43 (±0.74)	2.47 (±0.76)	2.39 (±0.78)	2.34 (±0.71)	2.36 (±0.69)
8	Average number of inseminations per pregnancy in primiparous cows (no.)	2.36 (±0.83)	2.36 (±0.89)	2.44 (±0.88)	2.48 (±0.98)	2.42 (±0.95)	2.32 (±0.87)	2.39 (±0.84)
9	Average number of inseminations per pregnancy in multiparous cows (no.)	2.39 (±0.71)	2.35 (±0.67)	2.42 (±0.81)	2.48 (±0.84)	2.37 (±0.86)	2.35 (±0.77)	2.33 (±0.77)
10	Average number of inseminations per pregnancy leading to a registered newborn (no.)^a^	0.66 (±0.58)	0.81 (±0.73)	0.67 (±0.64)	0.79 (±0.75)	0.67 (±0.63)	0.76 (±0.74)	0.65 (±0.61)
11	Total number of registered newborns (no.)^b^	5.04 (±4.18)	4.47 (±3.69)	5.34(±4.37)	4.74 (±3.80)	5.34 (±4.38)	4.71 (±4.22)	4.98 (±3.69)
12	Number of cows per farm (no.)	70.27 (±45.49)	70.54 (±48.23)	70.67 (±48.17)	71.52 (±50.00)	72.72 (±49.51)	73.73 (±52.68)	64.65 (±22.36)
13	Cows’ average age (months)	46.19 (±5.98)	46.66 (±6.67)	46.61 (±6.38)	46.73 (±5.98)	46.69 (±5.16)	46.77 (±5.18)	46.85 (±5.85)
14	Average length of dry period (days)	76.78 (±59.67)	76.03 (±66.95)	74.16 (±41.76)	74.15 (±38.01)	70.1 (±20.19)	67.96 (±13.57)	67.09 (±14.23)
15	Proportion of pregnant primiparous cows (%)	44.63 (±14.18)	45.74 (±15.34)	43.79 (±15.59)	45.96 (±15.99)	46.53 (±15.82)	46.48 (±16.22)	44.84 (±16.07)
16	Proportion of pregnant multiparous cows (%)	41.66 (±13.53)	42.21 (±14.07)	40.4 (±14.93)	43.13 (±14.41)	42.62 (±13.76)	42.42 (±14.75)	40.72 (±14.92)
17	Proportion of artificial inseminations (%)	91.67 (±20.39)	91.48 (±21.35)	91.61 (±22.42)	89.95 (±25.04)	89.02 (±26.32)	89.62 (±25.56)	89.65 (±25.39)
18	Proportion of culled cows (%)	2.54 (±3.09)	3.04 (±3.18)	2.27 (±2.29)	2.83 (±2.55)	2.37 (±2.40)	2.85 (±2.74)	2.31 (±2.35)
19	Cows’ average age at first delivery (months)	27.15 (±4.04)	27.87 (±6.67)	28.17 (±6.71)	31.32 (±39.57)	21.07 (±12.97)	20.9 (±12.00)	19.25 (±12.88)

^a^Calculated as the ratio between the average number of inseminations per pregnancy (indicator 7) and the total number of registered newborns (indicator 11) of the following 9^th^ month

^b^Includes only healthy calves that survived for at least 21 days after birth and the farmers were then obliged to register to the local health authority

The mixed-effects linear models showed that only two (out of the 11) performance indicators of interest varied significantly with respect to the reference semester (Jul-Dec 2011). These indicators were the average lactation length and the average calving-to-conception interval (Table 2). Except for the second semester of 2009, all other semesters showed a significantly longer lactation length – also known as days in milk (DIM) – than that of the reference semester (Table 2). Looking specifically at the same semesters over the years, herds’ lactation length was, on average, 6.3 days longer in the second semesters of 2010 compared to the reference semester (Table 2). Concerning the average calving-to-conception interval, a significant difference of approximately 4 days was observed between the second semester of 2009 only (LS-mean = 163.7 days) and the reference semester (LS-mean = 159.63 days) (Table 2).

**Table 2:** Adjusted estimates of the effects of semester on the average lactation length and average calving-to-conception interval of 127 dairy cattle herds of Treviso province, Italy, obtained from the final mixed-effects linear models with first order autoregressive covariance structure fitted to monthly measurements (January 2009 to June 2012)

Semester	Average lactation length (days)^a^				Average calving-to-conception interval (days)^b^			
	Least square mean†	Standard error	Coefficient	*p*-value	Least square mean†	Standard error	Coefficient	*p*-value
Jan-Jun 2009	203.50	2.13	7.65	0.0002	161.20	2.63	1.57	0.4448
Jul-Dec 2009	199.27	2.11	3.41	0.0671	163.72	2.62	4.09	0.0295
Jan-Jun 2010	202.21	2.03	6.36	0.0003	160.79	2.55	1.16	0.4923
Jul-Dec 2010	202.11	2.07	6.26	<0.0001	161.09	2.59	1.46	0.3077
Jan-Jun 2011	200.87	2.04	5.02	<0.0001	159.49	2.57	−0.14	0.8899
Jul-Dec 2011	195.85	2.12	Ref.	-	159.63	2.64	Ref.	-
Jan-Jun 2012	199.97	2.25	4.12	0.0042	159.98	2.72	0.35	0.7870

^a^Estimates are adjusted for variables 6 and 12–18 and for mean temperature (see Tables 1 and 3), all included as fixed effects. Random effects were set at the farm level and used sinusoidal terms for seasonal control

^b^Estimates are adjusted for variables 12–18, and for mean temperature (see Tables 1 and 3), all included as fixed effects. Random effects were set at the farm level and used sinusoidal terms for seasonal control

†Also referred to as marginal means, are the means after controlling for the other covariates included in the models

The effects of the control variables included in the final models for DIM and calving-to-conception interval are reported in Table 3. Except for the herd size and cows’ average age, all the other covariates in the final model for DIM were significantly associated with the outcome. An increase in the average monthly temperature, calving-to-conception interval, proportion of pregnant cows, and proportion of artificial (over natural) inseminations performed in the farm was significantly associated with an increase in DIM. Conversely, an increase in the length of the dry period and proportion of culled cows was associated with a decrease in DIM. Except for the herd size, cows’ average age, average monthly temperature and proportion of pregnant cows, all the other covariates in the final model for the calving-to-conception interval were significantly associated with the outcome and had associations similar to those found for DIM.

**Table 3:** Associations of the control variables included in the ‘semester-based’ mixed-effects linear models for the average lactation length and average calving-to-conception interval of 127 dairy cattle herds of Treviso province, Italy, with first order autoregressive variance structure fitted to monthly measurements (January 2009 to June 2012)

Control variable	Average lactation length (days)^a^			Average calving-to-conception interval (days)^a^		
	Coefficient	F-value	p-value	Coefficient	*F*-value	*p*-value
Herd size	−4.69	1.63	0.2047	−1.32	0.07	0.7858
Cows’ average age (months)	−0.09	0.51	0.4737	−0.19	2.21	0.1369
Average calving-to-conception interval (days)	0.15	85.63	<0.0001	-	-	-
Average monthly temperature	0.82	167.86	<0.0001	−0.05	1.25	0.2643
Proportion of pregnant primiparous cows (%)	0.33	213.63	<0.0001	−0.01	0.47	0.4928
Proportion of pregnant multiparous cows (%)	0.46	275.41	<0.0001	−0.07	9.21	0.0024
Average length of dry period (days)	−0.03	4.16	0.0414	−0.07	21.11	<0.0001
Proportion of artificial inseminations (%)	0.11	7.27	0.0071	0.43	120.17	<0.0001
Proportion of culled cows (%)	−0.45	61.78	<0.0001	−0.28	32.04	<0.0001

^a^Estimates are adjusted for semester (see Tables 2), included as fixed effect. Random effects were set at the farm level and used sinusoidal terms for seasonal control

The trimester-based analysis (Table 4) revealed that DIM and calving-to-conception interval were again the only indicators with significant variations as compared with the reference trimester (Oct-Dec 2011). The lactation length was, on average, 4.1 days significantly longer (*p* = 0.035) in Oct-Dec 2010 (LS-mean = 202.14 days) than in Oct-Dec 2011 (LS-mean = 198.00 days), but this was not the case (*p* = 0.193) in Oct-Dec 2009 (LS-mean = 200.96). Regarding the calving-to-conception interval, a difference of 7.7 days was observed between the last trimester of 2009 (LS-mean = 164.34 days) and the reference trimester (LS-mean = 156.63 days). The associations of the control covariates (Table 5) did not differ from those of the semester-based analysis.

**Table 4:** Adjusted estimates of the effects of trimester on the average lactation length and average calving-to-conception interval of 127 dairy cattle herds of Treviso province, Italy, obtained from the final mixed-effects linear models with first order autoregressive covariance structure fitted to monthly measurements (January 2009 to June 2012).

Trimester	Average lactation length (days)^a^				Average calving-to-conception interval (days)^b^			
	Least square mean†	Standard error	Coefficient	*p*-value	Least square mean^c^	Standard error	Coefficient	*p*-value
Jan-March 2009	200.27	2.33	2.27	0.3540	165.97	2.81	9.34	0.0004
Apr-Jun 2009	203.17	2.25	5.17	0.0303	160.03	2.75	3.40	0.1815
Jul-Sep 2009	197.72	2.28	−0.28	0.9052	162.91	2.77	6.28	0.0113
Oct-Dec 2009	200.96	2.27	2.96	0.1929	164.34	2.76	7.71	0.0012
Jan-March 2010	203.15	2.21	5.15	0.0212	162.01	2.70	5.38	0.0185
Apr-Jun 2010	201.95	2.18	3.95	0.0703	161.71	2.68	5.08	0.0199
Jul-Sep 2010	200.74	2.24	2.74	0.1935	162.40	2.73	5.77	0.0054
Oct-Dec 2010	202.14	2.24	4.14	0.0349	159.11	2.73	2.48	0.1921
Jan-March 2011	199.91	2.20	1.91	0.2916	158.10	2.71	1.47	0.3853
Apr-Jun 2011	199.82	2.22	1.82	0.2688	156.94	2.73	0.31	0.8338
Jul-Sep 2011	193.57	2.30	−4.43	0.0006	157.47	2.79	0.84	0.4645
Oct-Dec 2011	198.00	2.30	Ref.	-	156.63	2.79	Ref.	-
Jan-March 2012	200.70	2.38	2.70	0.0639	157.42	2.85	0.79	0.5395
Apr-Jun 2012	206.23	2.50	8.23	<.0001	158.22	2.91	1.59	0.3545

^a^Estimates are adjusted for variables 6 and 12–18 and for mean temperature (see Tables 1 and 5), all included as fixed effects. Random effects were set at the farm level and used sinusoidal terms for seasonal control

^b^Estimates are adjusted for variables 12–18, and for mean temperature (see Tables 1 and 5), all included as fixed effects. Random effects were set at the farm level and used sinusoidal terms for seasonal control

^c^Also referred to as marginal means, are the means after controlling for the other covariates included in the models

**Table 5:** Associations of the control variables included in the ‘trimester-based’ mixed-effects linear models for the average lactation length and average calving-to-conception interval of 127 dairy cattle herds of Treviso province, Italy, with first order autoregressive variance structure fitted to monthly measurements (January 2009 to June 2012)

Control variable	Average lactation length (days)^a^			Average calving-to-conception interval (days)^a^		
	Coefficient	*F*-value	*p*-value	Coefficient	*F*-value	*p*-value
Herd size	−4.53	1.51	0.2211	−1.34	0.08	0.7822
Cows’ average age (months)	−0.12	0.82	0.3643	−0.12	0.92	0.3381
Average calving-to-conception interval (days)	0.16	91.00	<.0001	-	-	-
Average monthly temperature	0.94	168.66	<.0001	−0.01	0.02	0.8754
Proportion of pregnant primiparous cows (%)	0.33	216.91	<.0001	−0.01	0.44	0.5086
Proportion of pregnant multiparous cows (%)	0.45	261.98	<.0001	−0.07	8.31	0.0040
Average length of dry period (days)	−0.03	3.25	0.0717	−0.07	23.67	<.0001
Proportion of artificial inseminations (%)	0.11	7.70	0.0056	0.42	114.97	<.0001
Proportion of culled cows (%)	−0.42	52.11	<.0001	−0.29	35.20	<.0001

^a^Estimates are adjusted for trimester (see Tables 4), included as fixed effect. Random effects were set at the farm level and used sinusoidal terms for seasonal control

## Discussion

This study assessed the potential impact of SBV emergence on a number of productive and reproductive performance indicators of dairy cattle herds in a SBV-affected province of North-East Italy, while accounting for a range of potential confounders, including weather conditions. Results indicated a statistically significant drop in the average lactation length and in the average calving-to-conception interval during the second semester of 2011, but none of the other indicators showed significant variations. These results were reproduced using a narrower temporal window based on trimesters.

A reduced number of DIM is strictly related to a decrease in the number of days between calving and conception, also known as days open, as cows that are re-impregnated early in lactation will enter earlier the dry period. Shortening the lactation period by means of early start of re-insemination (6–8 weeks postpartum) is a desirable target in modern dairy farm management [16–18]. Extended lactation periods are in fact an indicator of reproductive problems in the herd (e.g. decreased fertility, abortion, foetal resorption, non-return, etc.). The advantage of short lactation cycles is that more peaks of lactation will occur in a given time span, resulting in more milk yield and more calves delivered. Moreover, cows that are re-inseminated early in lactation perform better in terms of pregnancy rates [19]. Conversely, increased days open affect farm profitability via increased breeding costs coupled with reduced milk production due to the post-peak flattening of the lactation curve. Besides, increased days open in a previous parity have been associated with a higher risk of death and live culling around the subsequent calving, resulting in increased replacement costs [20]. It follows, therefore, that the significant decrease in DIM and days open observed here actually represents a positive outcome, which is not as such imputable to SBV emergence. If SBV would have had (detrimental) effects on these two indicators via, for instance, the onset of clinical disease, non-return and adverse pregnancy outcomes, an increase, rather than a decrease, in DIM and days open had to be expected. As it is highly unlikely that SBV improved the performance of dairy cattle herds, other factors than SBV are likely to be involved, e.g. the voluntary waiting period, artificial insemination technique, calving season (including changes in estrous synchronization in response to market needs), farm management policy, herd size, production level and parity [21], but also mere yearly variation. As some of these factors, such as the herd size, season, production level and parity were actually controlled for in our models, other (hitherto unknown) changes in farm management practices would probably explain the observed differences. Besides this, co-morbidities in the farms might have had an effect on the performance indicators. However, only three farms notified problems of mastitis caused by *Staphylococcus aureus* and *Streptococcus uberis*, but no other diseases were reported during the second semester of 2011, making concurrent health problems unlikely to have contributed significantly to the observed decrease in DIM and days open.

The main limitation of this study is that it was not possible to distinguish between “SBV-infected” and “SBV-free” herds, nor between different gradients of within-herd SBV prevalence. Rather, we assessed the overall impact of SBV based on a pre- and post-emergence design, assuming a homogeneous spread of infection across the study area. This assumption has some plausibility given the limited extension of the study area and the evidenced ability of SBV to spread rapidly and extensively [22]. Moreover, in the study area the prevalence of SBV in biting midges of the Obsoletus complex has been reported to be much higher (~10 times) than that of BTV [2]. Similar findings have been reported from other European countries [23, 24], suggesting a higher vector competence for SBV, which might counterbalance its shorter viraemia compared to BTV [1], a feature that draws further attention to the SBV potential for spreading. Accordingly, a model for between-farm SBV spread has shown that non-midge-borne transmission routes are unnecessary to explain the rapid spread of SBV [15]. Given that all herds in the study area were assumed to be equally exposed to SBV and effects at the herd level might be detectable at a finer scale depending on the within-farm prevalence, our results might be biased towards no significant effect because of insufficient insights into the spatial heterogeneity of SBV spread within the study area. Similarly, the use of relatively large temporal windows (semesters and trimesters), because of the difficulties in pinpointing the exact 1–2 week period in which animals were clinically ill, could have masked an actual effect. However, it can also be argued that any significant effect of SBV, including repercussions on farm profitability, would be better appreciable in a six to three month period interval, as finer temporal subdivisions would have complicated the interpretation of the results given the occurrence of small-scale punctual fluctuations in the (re)productive indicators. After all, a study in the Netherlands [11] was able to detect significance variations in herds’ performances attributable to SBV using a 3-month reference period.

As mentioned in the introduction, Veldhuis et al. [10] were able to demonstrate a significant, yet limited, reduction in milk production and calf mortality in the Netherlands during the hypothesized SBV transmission period by distinguishing between herds with high and low seroprevalence and between farms with and without reported clinical cases. The same approach allowed to highlight an increased rate of abortions, malformed lambs, dystocia and lamb mortality in SBV-positive versus SBV-negative sheep flocks in Belgium [12]. The lack of prevalence or clinical data for the herds under study was the main reason as to why we could not apply such an analytic approach, which was certainly likely to provide more insights in the impact of SBV. However, Veldhuis et al. [11] did not distinguish between herds based on their SBV infection levels, but performed separate analyses for the Dutch national population of dairy cattle herds and for the subgroup of herds reporting malformations in newborn calves, and found small but significant variations in several (re)productive parameters. Therefore, the present study can only make the point that, irrespective of the SBV infection levels and rates of clinical illness, overall at the province level the (re)productive performance of dairy cattle herds in the study area did not seem to have been significantly affected by the emergence of SBV, at least not in a way that there were detrimental effects on farm profitability. This is in line with the results of Wernike et al. [25] who monitored a farm near the city of Schmallenberg, Germany, between May 2011 and January 2012. All tested animals were SBV-positive after week 41 of 2011. Yet, no decrease in milk yield nor diarrhoea was observed, only transient fever. Moreover, no abortions, stillbirths or malformed calves were observed despite that at the end of September 2011 most cows were between 75 and 175 days of pregnancy, i.e. the critical gestation period. Similarly, the relatively low number of SBV-associated congenital malformations compared to the high level of SBV infection as indicated by seroprevalence studies suggest that these occur only rarely in calves [9]. Furthermore, calves infected *in utero* can clear SBV infection [26], and farms adopting calving patterns with the critical period falling mostly into periods of low vector activity (usually Dec-Mar) can experience lower impact [9]. Therefore, although it cannot be excluded that in some instances the impact of SBV may have been considerable at the individual farm level, it was not unexpected to find here that overall at the province level this impact did not appear to be significant. Our results agree, to a major extent, with those of a recent study on the impact of SBV on milk production, reproductive performance, and calf mortality in dairy cattle in Kleve district, Germany [11]. These authors could only find a small increase in the number of inseminations in the assumed SBV period, but no significant changes in calf mortality or milk production were found, indicating that SBV had a very limited impact, but also that there is considerable variation in the extent of such impact between countries.

Underreporting and under-ascertainment of SBV cases is known to be considerable [7]. This was especially true at the beginning of the epidemic, when the mild and transitory nature of the clinical disease, combined with the (initial) lack of diagnostic insights, led many cases to pass unnoticed. Even after the discovery of the virus and the launch of the international alert, many farmers might have not reported suspected cases because of the fear of bearing financial consequences. Thus, even if the reported cases could merely depict the enforcement of enhanced monitoring/control activities by local authorities, development of diagnostic tools and recognition of the lack of SBV zoonotic potential, a possible explanation for the absence of an evidenced negative effect of SBV in the study area may be due, to some extent, to the absence of reported clinical cases in 2011 [7, 9, 27].

The major impact of SBV originated from restrictions on international trade of susceptible animals, including semen and embryos [6]. Of course the rapid spread of the virus in a completely naïve host population resulted in some direct impacts. However, whether this scenario will happen again is a matter of discussion, especially since SBV is likely to remain endemically in Europe, allowing for herd immunity to persist [9]. New SBV outbreaks will therefore occur if the level of herd immunity declines or introductions of novel SBV strains occur. Nonetheless, based on our study, it seems that the future impact of SBV on dairy cattle will depend more on the trade restrictions applied than on the worsening of herds’ performance. It should be reminded, however, that our results are not based on empirical data on the exact SBV status of the herds, so we are likely to have underestimated the effect of SBV because the proportion of SBV-infected animals within a herd is variable and probably never 100 %. Moreover, the detrimental effects of SBV infection are only present during a limited period of gestation, and a large part of the animals in the herds may have been infected outside this critical period, having no detectable effect on performance.

## Conclusions

We assessed the potential impact of SBV emergence on several productive and reproductive performance indicators of dairy cattle herds in a SBV-affected province of North-East Italy, accounting for weather conditions and other herd-level factors that could also influence these indicators. Significant decreases in the average lactation length and calving-to-conception interval were observed during the likely SBV infection period. However, such decreases actually represent a positive outcome which is not imputable to SBV emergence *per se*, but rather mirrors other beneficial changes in farm management. While it is highly unlikely that SBV would have improved the performance of dairy cattle herds, the lack of significant variations in dairy herds’ (re)productive performance imputable to SBV is in line with the relatively mild expression of acute SBV infection in adult cattle. However, our results also contradict previous reports on reproductive problems, and may have underestimated the effect of SBV emergence since no data on the SBV status of the herds and animals therein were available to inform the analysis on the infection levels of the cattle population under study. Although the emergence of SBV might have significantly challenged some individual farms provided that most SBV infections had occurred in the critical gestation period, overall at the province level their (re)productive performances did not seem to have been significantly affected, at least not in a way that there were detrimental effects on farm profitability.

## Methods

### Study area and herd-level performance indicators

The study area consisted of the province of Treviso (2477 km^2^) in North-East Italy (Fig. 1). All dairy cattle farms subscribed to the Treviso’s Provincial Cattle Breeders Association (Italian acronym: APA) were included in the study. These were 127 intensive farms, with an average of 71 cows per farm (range 29–496) and a closed production system. These farms are under continuous monitoring of a number of productive and reproductive performance indicators by the APA, the membership of which is voluntary. APA members have access to several services aimed at improving livestock productivity and welfare. Such services include, among others, the monitoring of how farms perform, both in relation to their own historical records and for benchmarking against other farms. Herd-level monthly measurements of 19 (re)productive performance indicators of the abovementioned 127 farms were provided by the APA for the period between January 2009 and June 2012. These indicators, together with a summary of their values by semester, are reported in Table 1.

Indicators represent monthly averages (indicators 1–10, 13–15 and 19, see Table 1) or total monthly counts (indicators 11 and 12) based on the whole farm and are recorded automatically by a herd management software dedicated to the individual follow-up of every animal in the farm. Averages of milk production per cow and fat/protein milk content are determined in bulk milk or recorded individually at milking of each cow through their pedometers. The reproductive status of every animal at any point in time, including their inseminations, pregnancy diagnoses, deliveries, start and end dates of lactation and dry periods, is also individually recorded. Indicators are then automatically calculated by the APA based on the recorded data. Sporadically, because of various technical or logistical problems, some indicators are incalculable for specific farms at a given point in time; these missed observations are then treated as missing values. Abortions in these farms are notifiable to local veterinary services and are handled according to the regional control programme in force for bovine abortion [28].

### Weather data

Monthly mean temperature (°C) and relative humidity (%) data for the period between January 2009 and June 2012 were collected through 21 official weather stations distributed all over the province of Treviso. These data were provided by the Environmental Protection Agency of Veneto region (Italian acronym: ARPAV); a governmental agency with multiple missions related to environmental policy and research and is in charge, among other tasks, for the management of all weather stations of Veneto region. This includes collecting, validating, analyzing and disseminating meteorological data to external users. Each of the 127 farms included in the study was anchored to its closest weather station.

### Study periods

The overall study period spanned between January 2009 and June 2012. Significant variations in herds’ performance imputable to SBV were expected to be detectable during the second semester of 2011, which covers the most likely period of primary exposure to SBV in continental Europe [6, 9, 10, 15, 27]. Particularly, most ruminants in the study area were likely to have come into contact with the virus between October and December 2011. This was indicated by the detection in the study area of the first case of SBV-induced foetal malformations in the full-term dystocic goat at the beginning of February 2012 [8], as the most susceptible gestation period in ovicaprines is between 45 and 60 days of pregnancy [9]. Moreover, virus detection in midges has demonstrated that SBV had been circulating in the study area since September 2011, yet any circulation before then was deemed unlikely [2]. Furthermore, a convenience sample of 487 sera collected in January and in March 2012 at 14 cattle farms in the study area within the framework of statutory surveillance activities for brucellosis and bovine leukosis, were tested for SBV at the Istituto Zooprofilattico Sperimentale delle Venezie using the commercial ID Screen® Schmallenberg virus competition multi-species ELISA test (ID.vet Innovative Diagnostics, France), according to manufacturer’s instructions. Serological testing revealed an overall positivity rate for SBV of 76 % (range 30-100 %, unpublished data). Although no sera collected in 2011 were available for SBV testing, this, together with the above-mentioned viral detections in midges and animals in 2011, provided evidence that exposure to SBV was already serologically detectable at the earliest beginning of 2012, supporting the notion that SBV had shortly before spread over the study area, i.e. in the last semester of 2011, and most likely in Oct-Dec 2011. Therefore, the second semester of 2011 and particularly the last trimester therein were taken as the reference periods (i.e. the SBV infection periods) for assessing the possible impact of SBV (see below).

A putative effect of the clinical disease on the productive parameters (i.e. milk production and milk protein and fat contents) and on the reproductive parameters regarding immediate fertility of the conception frame (i.e. number of lactations, lactation length, calving-to-conception interval, number of inseminations per pregnancy - since pregnancies would already have occurred during the acute phase of the infection) was indeed expected to occur in the SBV infection periods. Only the reproductive indicators related to newborns (e.g. number of inseminations per pregnancy leading to a healthy newborn and total number of healthy newborns) were lagged 9 months later in time, as they would have been affected by the intrauterine infection rather than the acute (symptomatic) phase.

Given the limited extension of the Treviso province, all cattle herds in the study area were assumed to have been exposed to SBV. This was supported by the extraordinary ability of SBV to spread rapidly over large geographical areas, as demonstrated by several seroprevalence studies indicating that virtually every cow housed in SBV-affected areas had come into contact with the virus [22, 29–31]. As neither prevalence nor clinical data on SBV were available for the herds in question, this study could only assess whether there were significant variations in province-level (re)productive performances of dairy cattle herds imputable to the emergence of SBV, regardless of their within-herd prevalence and rate of clinical illness.

### Data analysis

Each of the first 11 performance indicators listed in Table 1 was modelled, as dependent variable, using mixed-effects linear models for longitudinal data, given that repeated measurements were made on the same farms over time; a random effect at the farm level was included in all models. The other eight indicators listed in Table 1, together with the mean temperature and relative humidity, were considered for inclusion as control variables in the models. Except for herd size, which is a dichotomous variable of large *vs.* small farms, all other control variables were treated as continuous covariates. Analyses were performed using the procedure PROC MIXED in SAS v.9.3, with the RANDOM and REPEATED statements to model the random effect at the farm level and the multiple observations made over time on the same farms. Variables were selected based on previous studies, biological plausibility of being influenceable by SBV, and scientific interest of the research team. Collinearities between variables were checked by looking at their correlation matrix (Additional file 1), and selection between collinear variables was based on the improvement in model fit as revealed by the Akaike information criterion (AIC) values.

The independent variable of interest to describe the average variation in herds’ performance over the study period was the semester, which was included as categorical fixed effect (Jan-Jun 2009, Jul-Dec 2009, Jan-Jun 2010, Jul-Dec 2010, Jan-Jun 2011, Jul-Dec 2011, Jan-Jun 2012), with the semester Jul-Dec 2011 (SBV infection period) being the reference category. Further models were built narrowing the SBV infection period by splitting the above semesters in trimesters as to assess variations in herds’ performance relative to the reference trimester Oct-Dec 2011. All comparisons focused on the same periods of the years 2010 and 2009.

To control for unobserved covariates with a systematic behaviour in time, sinusoidal terms were included in the models as random effects. The structure for the random effect was selected using restricted maximum likelihood (REML) estimation, by comparing the −2 REML log-likelihood for the reference model with the −2 REML log-likelihood of the nested model [32]; all random effects were significant. Several covariance structures (autoregressive, compound symmetry, and unstructured) were assessed to model the residuals associated with multiple observations originating from the same farm, and the first order autoregressive covariance structure was identified as the best one to capture the within-herd correlation.

Non-significant (*p* > 0.05) fixed effect terms were dropped from the models in stepwise backward fashion, after having evaluated the results of Type III F-test. Additionally, the effect of removing variables on the other covariates included in the models was also monitored, and variables causing a significant change in the other covariates when removed were retained in the model to control for their effect regardless of significance. Normality of residuals was checked using normal probability plots to confirm absence of any remaining structure not accounted for by the models. For the sake of simplicity, only the final models of those performance indicators showing significant variations compared to the reference semester were presented. Results were expressed as adjusted means (LS-means) and standard errors. The basic equation of the regression models with *n* candidate independent variables (*n* = 1, 2…21) used in this study was as follows:

$$ E\left({y}_{ji}\right)={\beta}_0+{\beta}_1{X}_{1j}+{\beta}_2{X}_{2t}+{\beta}_3{X}_{3ij}\dots +{\beta}_n{X}_{nij}+{u}_{oj}+{u}_{1i} \sin \left(2\pi \times \mathrm{mont}{\mathrm{h}}_i/12\right)+{u}_{2i} \cos \left(2\pi \times \mathrm{mont}{\mathrm{h}}_i/12\right)+{\varepsilon_{\mathit{\mathsf{j}}}}_{\mathit{\mathsf{i}}} $$

where *E*(*y*_*ji*_) is the expected value of the dependent variable in herd *j* (*j* = 1, 2…127) and month *i* (*i* = 1, 2…42). The parameter *β*_1_ is the fixed effect regression coefficient of the independent variable *X*_1*j*_ denoting the size of the *j*^th^ herd; *β*_2_ is the fixed effect regression coefficient of the independent variable *X*_2*t*_ specifying the *t*^th^ semester (*t* = 1, 2…7) or the *t*^th^ trimester (*t* = 1, 2…14) of observation; *β*_3_… *β*_*n*_ are the fixed effect regression coefficients of the *n* herd- and month-varying independent variables *X*_3*ij*_… *X*_*nij*_ as reported in Table 1, including the weather variables; *u*_0*j*_ is the random effect associated with the intercept for herd *j*, and *u*_1*i*_ and *u*_2*i*_ are the sine and cosine random effects for month *i*, respectively; *ε*_*ji*_ is the random error term for herd *j* in month *i*.

## Ethics statement

Data on cattle performance were collected by the Treviso’s Provincial Cattle Breeders Association (APA) among its member farms as part of their regular monitoring activities of productive and reproductive performance indicators to assess how the farms perform in relation to their own historical records and for comparison to other farms. Data were automatically obtained from bulk milk, at milking via cows’ pedometers, and/or by veterinarians during routine reproduction management practices in cattle following standards of good veterinary practice in Italy and in the EU. No ethical approval was required for this study.

## Additional file

Additional file 1:
**Correlation matrix of the continuous productive and reproductive performance indicators of dairy cattle herds.** (DOCX 18 kb)
