# Supplementary material for: Productive and reproductive performances of dairy cattle herds in Treviso province, Italy (2009–2012): an assessment of the potential impact of Schmallenberg virus epidemic
Source: BMC Vet Res. 2015 Aug 11;11:193. doi: 10.1186/s12917-015-0527-1 (PMC4531501; doi:10.1186/s12917-015-0527-1)
Supplement: Additional file 1: — Correlation matrix of the continuous productive and reproductive performance indicators of dairy cattle herds. (DOCX 18 kb) [file 12917_2015_527_MOESM1_ESM.docx]

**Supplemetary Material.** Correlation matrix of the continuous productive and reproductive performance indicators of dairy cattle herds.

| **Pearson’s Correlation Coefficient  Prob > \|r\| with H_0_: Rho=0  Number of observations** | | | | | | | | |
| --- | --- | --- | --- | --- | --- | --- | --- | --- |
|  | **Cows' average age (months)** | **Average calving-to-conception interval (days)** | **Average monthly temperature** | **Proportion of pregnant primiparous cows (%)** | **Proportion of pregnant multiparous cows (%)** | **Average length of dry period (days)** | **Proportion of artificial inseminations (%)** | **Proportion of culled cows (%)** |
| **Cows' average age (months)** | - | 0.09222  <0.0001  4662 | -0.03078  0.0380  4545 | -0.03258  0.0261  4662 | -0.06227  <0.0001  4662 | 0.14490  <0.0001  4662 | -0.17404  <0.0001  4662 | -0.05758  <0.0001  4662 |
| **Average calving-to-conception interval (days)** | 0.09222  <0.0001  4662 | - | -0.02120  0.1530  4545 | 0.06866  <0.0001  4662 | 0.08350  <0.0001  4662 | -0.05559  0.0001  4662 | 0.28401  <0.0001  4662 | -0.02779  0.0578  4662 |
| **Average monthly temperature** | -0.03078  0.0380  4545 | -0.02120  0.1530  4545 | - | 0.28702  <0.0001  4545 | 0.53745  <0.0001  4545 | 0.02489  0.0934  4545 | 0.00693  0.6402  4545 | -0.05992  <0.0001  4545 |
| **Proportion of pregnant primiparous cows (%)** | -0.03258  0.0261  4662 | 0.06866  <0.0001  4662 | 0.28702  <0.0001  4545 | - | 0.47409  <0.0001  4662 | 0.04614  0.0016  4662 | 0.18006  <0.0001  4662 | -0.07577  <0.0001  4662 |
| **Proportion of pregnant multiparous cows (%)** | -0.06227  <0.0001  4662 | 0.08350  <0.0001  4662 | 0.53745  <0.0001  4545 | 0.47409  <0.0001  4662 | - | 0.01449  0.3227  4662 | 0.20745  <0.0001  4662 | -0.07532  <0.0001  4662 |
| **Average length of dry period (days)** | 0.14490  <0.0001  4662 | -0.05559  0.0001  4662 | 0.02489  0.0934  4545 | 0.04614  0.0016  4662 | 0.01449  0.3227  4662 | - | 0.02046  0.1625  4662 | -0.01210  0.4088  4662 |
| **Proportion of artificial inseminations (%)** | -0.17404  <0.0001  4662 | 0.28401  <0.0001  4662 | 0.00693  0.6402  4545 | 0.18006  <0.0001  4662 | 0.20745  <0.0001  4662 | 0.02046  0.1625  4662 | - | -0.01155  0.4305  4662 |
| **Proportion of culled cows (%)** | -0.05758  <0.0001  4662 | -0.02779  0.0578  4662 | -0.05992  <0.0001  4545 | -0.07577  <0.0001  4662 | -0.07532  <0.0001  4662 | -0.01210  0.4088  4662 | -0.01155  0.4305  4662 | - |
